# Genetic engineering of sex chromosomes for batch cultivation of non-transgenic, sex-sorted males

**DOI:** 10.1371/journal.pgen.1009180

**Published:** 2020-11-02

**Authors:** Siba R. Das, Maciej Maselko, Ambuj Upadhyay, Michael J. Smanski

**Affiliations:** 1 Department of Biochemistry, Molecular Biology, and Biophysics, Saint Paul, MN, United States of America; 2 Biotechnology Institute, University of Minnesota, Saint Paul, MN, United States of America; HudsonAlpha Institute for Biotechnology, UNITED STATES

## Abstract

The field performance of Sterile Insect Technique (SIT) is improved by sex-sorting and releasing only sterile males. This can be accomplished by resource-intensive separation of males from females by morphology. Alternatively, sex-ratio biasing genetic constructs can be used to selectively remove one sex without the need for manual or automated sorting, but the resulting genetically engineered (GE) control agents would be subject to additional governmental regulation. Here we describe and demonstrate a genetic method for the batch production of non-GE males. This method could be applied to generate the heterogametic sex (XY, or WZ) in any organism with chromosomal sex determination. We observed up to 100% sex-selection with batch cultures of more than 10^3^ individuals. Using a stringent transgene detection assay, we demonstrate the potential of mass production of transgene free males.

## Author summary

In this manuscript, we describe and demonstrate in a model system a new genetic engineering approach for producing sex-sorted, non-transgenic batches of an organism with genetic sex determination. This approach could impact pest control programs using Sterile Insect Technique.

## Introduction

Insect pests impose a major burden to food production and human health worldwide. The most successful population control method in use today is the sterile insect technique (SIT)[1]. SIT relies on mass rearing of pest insects followed by a sterilization treatment (*e*.*g*. X-ray irradiation). Sterilized insects are released into the wild where sterile males compete with wild males to mate with wild females. Since females of many pest insects only mate once in their lifetime, mating with a sterile male prevents successful reproduction. Sufficiently large releases of sterile insects can be used to eliminate wild populations or prevent their establishment in a new area[2]. Also, SIT is considered safe to humans and the environment, as there are less off-target impacts compared to the application of chemical pesticides.

Existing SIT programs are used to control several major agricultural and livestock pests including the New World Screwworm (*Cochliomyia hominivorax*)[3], Mediterranean Fruit Fly (*Ceratitis capitata*)[4], and Queensland Fruit Fly (*Bactrocera tryoni*)[5]. All together, these programs produce and release billions of sterile insects on a weekly basis. The application of SIT to control the New World Screwworm resulted in the eradication of this livestock pest in North and Central America, with an estimated value of over $US 1 Billion/year[3].

SIT for many insects, including *C*. *hominivorax* and *B*. *tryoni*, currently involves releasing both sterilized males and females. However, the effectiveness of SIT for any target pest can be substantially increased if only males are released since they will then seek out wild females instead of mating with co-released sterile females[6]. In some insect pests, such as the Yellow Fever Mosquito (*Aedes aegypti)*, it is crucial that SIT programs only release males since sterile females may transmit pathogens.

A variety of sex-sorting techniques have been developed[7]. Mechanical separation of *Aedes aegypti* pupae based on size differences[8] can be effective and flow cytometric separation of transgene expressing female *Anopholes gambiae* has been demonstrated[9], however, these approaches can be labor intensive or require sophisticated equipment. Combining irradiation with temperature sensitive lethal (*tsl*) mutant strains of *C*. *capitata* is presently in use SIT programs[10]. Repressible transgenic female-elimination constructs act as genetic control systems on their own and have been developed for *Ae*. *aegypti*[11], *C*. *hominivorax*[12], Sheep Blow Fly (*Lucilia cuprina*)[13], Diamondback moth (*Plutella xylostella*)[14], Pink Bollworm (*Pectinophora gossypiella*)[14], and Silkworm (*Bombyx mori*)[15]. Public resistance and regulatory hurdles have unfortunately limited the broad use of released transgenic insects for pest control.

**Fig 1:**
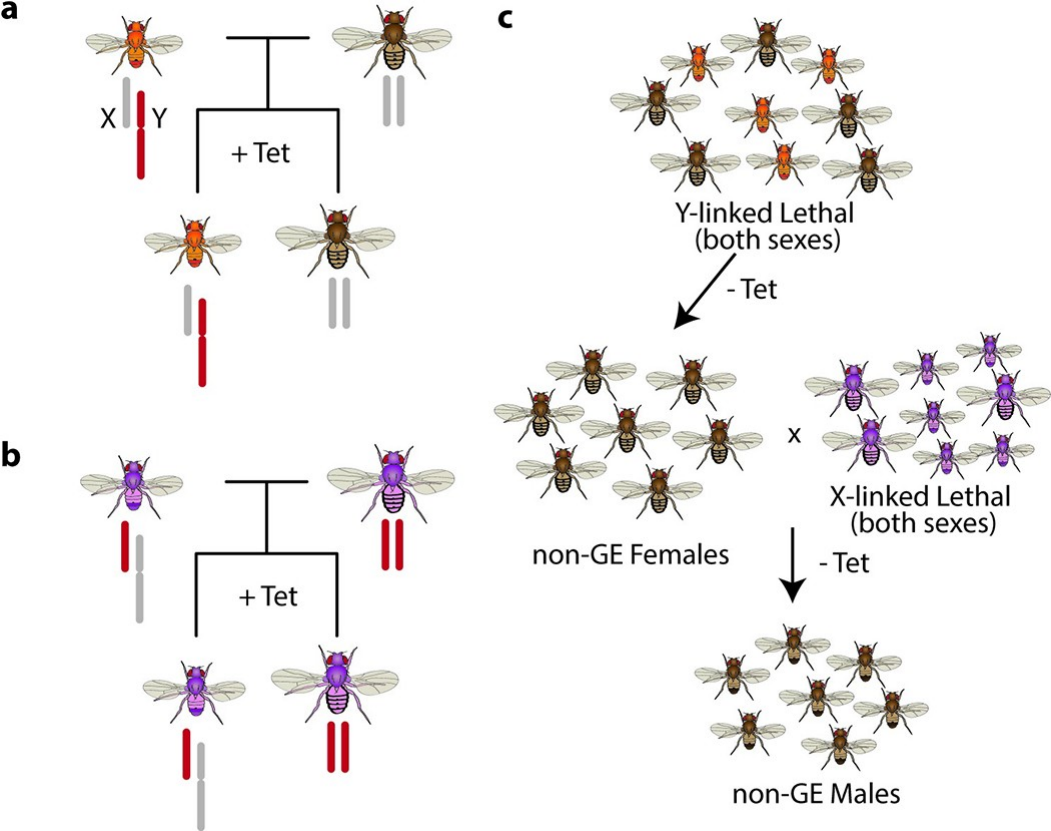
Overview of STSS. Minimal requirements for each strain to be used in STSS, including true-breeding population with conditional Y-linked lethality (**a**) or conditional X-linked lethality (**b**). (**c**) Mating scheme in absence of lethal gene repressor. Combining non-transgenic females produced from the Y^L^ strain with adult flies from the X^L^ strain results in death of all offspring except for non-transgenic males. This includes offspring from mating events of X^L^ males and females. Tet, tetracycline.

We describe here Subtractive Transgene Sex Sorting (STSS) which is a genetic approach to produce non-transgenic males. STSS relies on two transgenic strains; each of which has an embryonic or larval lethal genetic circuit that can be repressed (**Fig 1a**). One of the strains has the lethal circuit on the Y-chromosome (Y^L^ strain) and the other has the lethal circuit on the X-chromosome (X^L^ strain). Non-transgenic males are produced in a two-step mating scheme (**Fig 1b**). First, the Y^L^ strain is grown in media that activates the lethal circuit, resulting in non-transgenic females in the subsequent generation. These non-transgenic females are combined with the X^L^ strain in media that activates the lethal circuit. Mating between the X^L^ males and non-transgenic females results in non-transgenic males. All other offspring die from expression of the X-linked lethal circuit. This technique is transferable to any organism that relies on genetic, as opposed to environmental, sex determination[16].

## Results

### Design and construction of a repressible lethal transgenic construct

**Fig 2:**
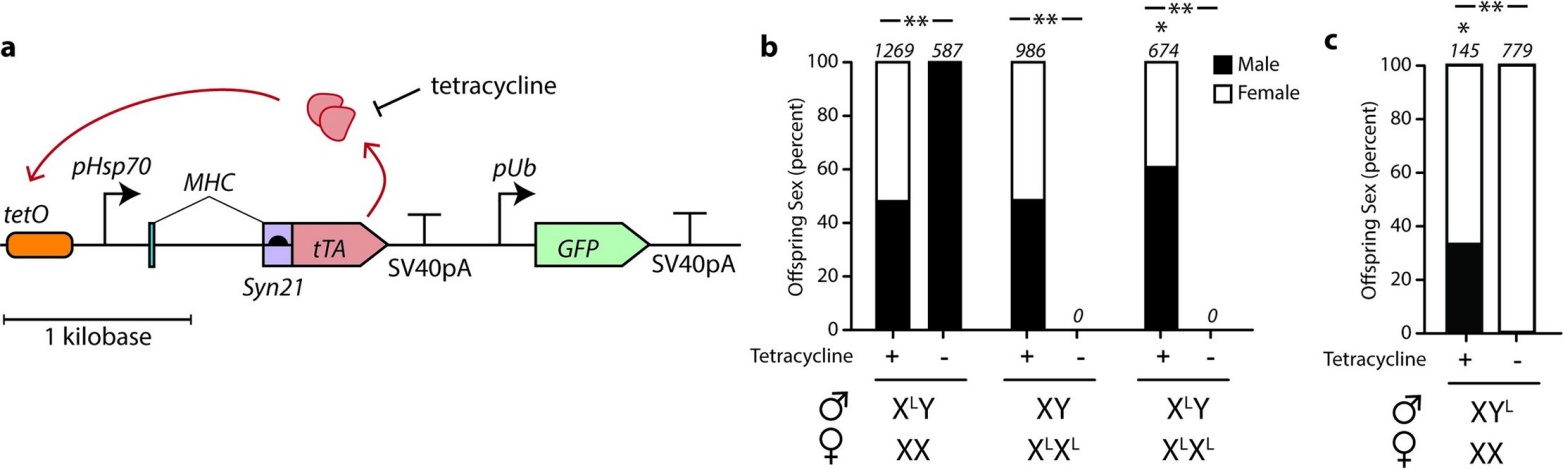
Sex-chromosome linked tet-repressible lethal circuits are effective. (**a**) Construct-level schematic of tet-repressible lethal circuit used in this study. (**b**) Proportion of male and female offspring generated from self-mating of DmX^L-tTA^ or with non-transgenic (*w*^*1118*^) flies in presence or absence of tetracycline. Genotypes of parental files are indicated below x-axis. Numbers above bars indicate total number of progeny produced from six biological replicates. (**c**) Proportion of male and females generated from mating DmY^L-tTA^ with non-transgenic (*w*^*1118*^) flies in presence or absence of tetracycline. Numbers above bars show total number of progeny produced from three biological replicates. * indicates statistically significant difference from expected 50:50 male:female sex ratio (chi-squared test, p < .05). ** indicates a statistically significant difference between the +tet and -tet groups (chi-squared test, p<0.001).

A repressible lethal genetic construct can be designed with a conditionally-expressed promoter driving a toxic gene product. We selected a tetracycline-repressible *hsp70* minimal promoter (*pHsp70*) due to its well-characterized behavior in model and applied insect species[13,17]. To drive lethality, we expressed the tet-transactivator (tTA), whose VP64 transactivation domain is toxic to cells when strongly expressed. We made tTA expression constructs with a positive feedback loop wherein *pHsp70* drives basal expression of tTA similar to what has been previously described[18,19] (**Fig 2a**). The presence of tetracycline prevents transactivation by tTA and keeps the construct expressed at basal, sub-lethal levels. In the absence of tetracycline, tTA binds to operators upstream of *pHsp70* and establishes a positive feedback loop which generates lethal amounts of tTA. We incorporated an MHC intron and syn21 5'-UTR translational enhancer features[19] to further boost tTA expression. Tuning gene expression in lethal transgenic constructs is a balance between (i) incurring fitness effects from leaky expression in the repressed ‘off’-state and (ii) incomplete penetrance due to weak expression in the de-repressed ‘on’-state. Our priority was to ensure complete penetrance of the lethal phenotype in the derepressed state, so we designed the construct to favor strong expression since the transgene will be absent in the sorted males.

To test the genetic circuit design, we engineered *D*. *melanogaster* due to its powerful genetic toolkit and also because its serves as a model for other insect pests. We used ΦC31-mediated transgenesis to integrate a single copy of the tTA circuit into *att*P landing sites on the X and Y chromosomes of two separate strains (**Fig 2b**, referred to as DmX^L-tTA^ and DmY^L-tTA^ from here on). Both of these strains were maintained in the presence of 100 μg/ml tetracycline. The genotype of transgenic flies was confirmed by PCR amplification. The DmX^L-tTA^ were mated with *FM6*, an X-chromosome balancer strain, and then selfed to screen for females homozygous for the modified X-chromosome, which was confirmed by PCR. From this point on, DmX^L-tTA^ and DmY^L-tTA^ were maintained as true-breeding lines in the presence of tetracycline.

### Performance of repressible lethal genetic constructs

To test the efficiency of toxic gene expression, virgin females and males (three each) were mated on media lacking tetracycline. In each of at least three replicate crosses, no DmX^L-tTA^ offspring survived to adulthood (**Fig 2b**). This suggests that the repressible lethal transgenic construct is sufficiently strong to cause lethality in two copies (females) or one copy (males). In an analogous experiment with DmY^L-tTA^ flies, ten replicate crosses produced a total of 777 females and only two males (99.7% female). These males did not reproduce when subsequently mated with non-transgenic females and lacked a Y chromosome (XO; **S2 Fig**), likely a result of nondisjuction. Thus, both DmX^L-tTA^ and DmY^L-tTA^ produced a sufficiently lethal phenotype in the absence of tetracycline to remove the transgene from the accessible gene pool (**Fig 2b and 2c**). There was however a background toxicity of the tTA circuit which resulted in a statistically significant (p < 0.05) but not substantial impact of male to female ratio in both DmX^L-tTA^ and DmY^L-tTA^ lines (**Fig 2b and 2c**).

### Sub-stoichiometric ratio of mixed-sex DmXL^tTA^ to female DmYL^tTA^ sufficient for non-transgenic male production

**Fig 3:**
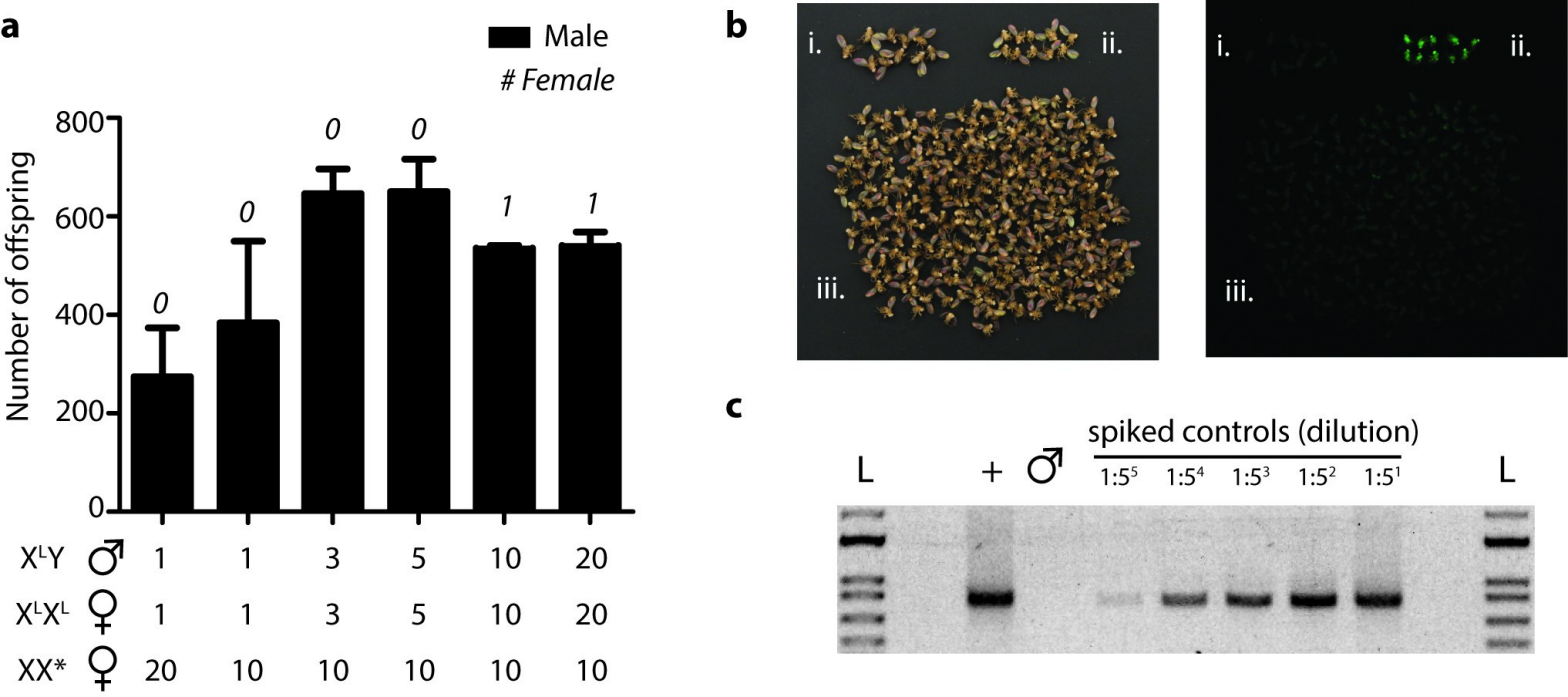
Batch production of adult males via STSS. (**a**) Average number of adult males obtained from mating between different proportions of non-transgenic female flies obtained from DmY^L-tTA^ in absence of tetracycline (‘XX*’) when combined with adult DmX^L-tTA^ flies in absence of tetracycline. Data represent mean numbers from 2–3 of biological replicates with error bars showing standard deviation. Average numbers of females produced are indicated numerically above bars. Numbers below x-axis indicate ratio of genotypes in parental generation. Total numbers from all replicates are (from left to right): N = 822, N = 1151, N = 1292, N = 1301, N = 1086, N = 1078 (**b**) Bright-field (left) and fluorescent (right) images of parental (i) 10 DmY^L-tTA^ females produced in the absence of tetracycline (‘XX*’), parental (ii) 10 DmX^L-tTA^ males, and (iii) approximately 400 offspring from the batch production of non-transgenic males. Files in (i) and (ii) are only included for visual comparison to the STSS males; they were not present in the final batch of STSS flies. (**c**) PCR amplification of transgene cassette from genomic DNA isolated from 10 DmX^L-tTA^ flies (+), 1180 batch-produced STSS males (male symbol), or STSS gDNA spiked with DNA from DmX^L-tTA^ flies at 5-fold dilutions from 1:5 (right) to 1:3125 (left). L denotes 1 kb plus DNA ladder (Thermo Fisher Scientific, Waltham, MA).

Non-transgenic males can be generated by crossing non-transgenic females produced by the DmY^L-tTA^ strain and males from a mixed-sex true-breeding population of DmX^L-tTA^ flies (**Fig 1c**). We hypothesized that the number of non-transgenic males produced will be directly related to the number of non-transgenic mothers, but robust to decreasing numbers of DmX^L-tTA^ fathers. This would be important for economically scaling-up the production of non-transgenic males for SIT programs. We performed experimental crosses between non-transgenic females and DmX^L-tTA^ mixed-sex populations to determine the minimum sufficient ratio of parental genotypes. We observed a monotonically increasing number of total offspring produced as the ratio of DmX^L-tTA^ males to DmY^L-tTA^ females increased from 1:20 to 3:10 (**Fig 3a**). The offspring number appeared to plateau or even decline after further increasing the number of DmX^L-tTA^ males. This suggests that a ~1:3 ratio is sufficient to ensure that the number of DmX^L-tTA^ males are not limiting the total number of offspring produced.

At or below the optimal ratios of DmX^L-tTA^ males to DmY^L-tTA^ females, we observed 100% male offspring (N_combined_ = 5388 male offspring, 0 female offspring). We observed a total of 4 female offspring across all replicates when the ratio of DmX^L-tTA^ males to DmY^L-tTA^ females was 10:10 or 20:10 (N_combined_ = 2142 male offspring, 4 female offspring). It is unclear how these females were able to survive, but they lacked a GFP phenotype, did not appear to be transgenic, and did not carry a Y chromosome (**S2 Fig**), indicating that they were not XXY females.

### Large-scale cultivation of non-transgenic males suitable for egg release

Next we tested the effectiveness of producing only non-transgenic males by batch cultivation, following the mating scheme in **Fig 1c**. We transferred a true-breeding culture of DmY^L-tTA^ to media lacking tetracycline and cleared all adults after 24 hours. The resulting offspring from the tetracycline-free medium were mixed at a 2:1 ratio with adults from a true-breeding population of DmX^L-tTA^ flies. Adults from this cross were cleared after 3 days. This mating yielded 2932 males (N = 3, 977±144) and one female. None of the >1000 males screened contained the GFP transgene marker. To ensure the lack of GFP detection (**Fig 3b**) was not due to transgene silencing, we isolated genomic DNA from more than 1000 male STSS flies and screened for presence of the transgene by PCR (expected fragment size of 821 bp). We observed a clear band of the expected size from a positive control (gDNA isolated from 10 DmX^LtTA^ flies) but not in gDNA isolated from the putative non-transgenic males (**Fig 3c**). Spiking trace amounts of positive control gDNA confirmed that limit of detection via this assay at less than 1:3000 transgenic:non-transgenic gDNA. This confirmed that the assay was sufficiently powerful to detect any transgenic flies that would have been present in the screened population.

## Discussion

This study demonstrated the proof of concept for STSS using *D*. *melanogaster* as a model system. The basic genetic parts have been demonstrated in numerous pest insects and we believe that STSS can be readily adapted to improve SIT programs by enabling efficient sex-sorting for male only release. We show here that STSS can be implemented with a variety of lethal genetic constructs, including female-lethal constructs, provided they are localized to the appropriate sex chromosome (**S1 Fig**). Although we used ΦC31 mediated integration, recent advancements with CRISPR systems have found that it’s possible to target integration to many genomic loci in insects, including the repeat rich Y chromosome[21]. Our approach allows for generation of transgene-free males in species where males are heterogametic. For species where the female is heterogametic (i.e. lepidoptera), generation of transgene-free males is simplified and would only require a W^L^ construct. In theory, this approach could be applied to species with homomorphic sex chromosomes, assuming the sex-determining region of the sex chromosome is accessible to transgene integration technology.

We did not observe any transgenic flies that were able to reproduce in the absence of tetracycline. However, the limit of detection of the scale up of our experiments only allow us to state that the transgenic fly escape is less than 0.1% (**Fig 3c**). SIT programs generate millions of animals for release on a weekly basis and it is possible that some small number of transgenic animals would be released via the STSS method. USDA Organic certification in the United States prevents organic farms from utilizing GMO technology, but does not establish a detection threshold. The Non-GMO project, a leading certifier of non-GMO status, establishes transgene threshold levels of 0.25% to 1.5% depending on the product category[22]. Thus, our application of STSS in *D*. *melanogaster* produces males that could in principle be certified as non-GMO.

Sex sorting for current SIT programs at mass rearing facilities is done by a combination of genetic and phenotypic features. By far the most commonly used sex sorting method is via a *tsl* mutation that makes females sensitive to elevated temperature[23]. The problem with using *tsl* mutations is that the strains are semi-sterile due to chromosomal translocation. To produce same number of males they require at least three times as large colony as their wildtype counterparts. We did not observe a substantial decrease in the number of males produced from our STSS approach, although this would need to be confirmed at a mass-rearing scale. Alternatively, sexes can be separated based on morphology. We have not compared the economics of maintaining two unique strains during mass-rearing operations to that of separating sexes based on morphology, but that would be an obvious consideration before adopting this approach for biocontrol programs.

By design, males produced by STSS are not sterile and hence would require combining with means to induce sterility, such as radiation treatment before they can be released for biocontrol. There are several SIT programs in place whose efficacy suffers from release of both male and female irradiated insects. We envision STSS being integrated with a traditional SIT campaign to increase the efficacy while remaining ‘non-GMO’. The small number of females produced via STSS (0.1%) would not substantially decrease the performance of a SIT campaign.

While this approach could also be coupled with genetically engineered incompatibility for biocontrol, doing so would no longer be considered ‘non-GMO’. This could still be an advantageous approach for gaining regulatory approval. Federal regulators consider each transgene as an ‘active ingredient’, and removing the sex-sorting transgenic element via STSS would produce a male-only, genetically incompatible strain with one less transgenic component. Our approach would require further optimization to be suitable for combining with *Wolbachia*-mediated Insect Incompatibility Technique (IIT), as this method is especially sensitive to even low numbers of female release.

The observed sex-ratio bias of the two strains used in the study indicates the toxicity of the transactivator in the lethal circuit. The altered sex ratio however has minimum impact on the final males produced by the system, since most of the mating is done in the absence of tetracycline where insects of only predicted sex emerge. The demonstrated male accuracy rate of 99.9% is much better than what is routinely observed in mass rearing facilities[24]. The occasional emergence of females in the final mating appeared to not contain the Y chromosome nor the genetically modified X chromosome, suggesting a possible role of non-disjunction.

STSS is based on a genetic design already demonstrated in many insect species. However, it is possible that the lethal circuits could not be inserted into sex chromosomes of some species with current genetic tools. STSS will not be useful in those species. During mass-rearing, the lethal circuit may accumulate mutations that render it inactive. Implementation of this approach in a mass rearing environment should be coupled with a genetic monitoring program to identify and mitigate such events. Encouragingly, there does not appear to be a fitness or fecundity phenotype during normal propagation of our engineered insect lines. More experiments are needed to understand the long-term stability of these genetic constructs under mass rearing conditions. These would be more relevant in an applied biocontrol species than in a model system like *D*. *melanogaster*. One option for minimizing the likelihood of genetic breakdown is to link the lethal circuit to an essential gene, which would eliminate the mutant allele from the gene pool. Alternatively, redundant lethal circuits that employ different mechanisms of lethality could increase the evolutionary robustness of this approach[25,26]. Leveraging inducible systems that do not require tetracycline addition may be preferable provided they can achieve similar levels of control over transgene expression. Tetracycline is known to alter insect microbiomes, but the impact this would have on mating success is still unclear and would need to be examined more closely on a case-by-case basis[27]. Although STSS requires tetracycline for the maintenance of two strains, much of the rearing meant for field release would be done in absence of tetracycline.

## Materials and methods

### Plasmid construction

The tetracycline repressible lethal circuit was made by adapting a previously described female-lethal piggybac vector, pB[FL3][13]. We replaced the female specific intron and 5'-UTR with a myosin heavy chain intron and syn21 translational enhancers[20]. The final plasmid, pMM7-10-1, was made by transferring the lethal circuit to pUB-EGFP[28] which contains an *attB* site for ΦC31 mediated integration and ubiquitin promoter driven EGFP expression.

The tetracycline female lethal circuit was made by adapting a previously described female-lethal piggybac vector, pB[FL3][17]. The final plasmid, pMM7-8-1, was made by transferring the lethal circuit to pUB-EGFP[28], which contains an *attB* site for ΦC31 mediated integration and ubiquitin promoter driven EGFP expression. Annotated plasmid sequences for pMM7-10-1 and pMM7-8-1 have been deposited in GenBank with accession numbers MN630870 and MN630871, respectively.

### Generating and maintaining transgenic drosophila strains

*D*. *melanogaster* strains were maintained at 25°C and 12 h days in cornmeal agar (NutriFly, Genesee Scientific, San Diego, CA) supplemented with 10–200 μg/ml tetracycline, as necessary. Transgenic repressible lethal *D*. *melanogaster* strains where generated by microinjection (Bestgene Inc, Ca) and ΦC31 mediated integration of pMM7-10-1 into the X-chromosome *attP* site of y[1] w[*] P{y[+t7.7] = CaryIP}su(Hw)attP8 (BDSC #32233)[29] to make DmX^L-tTA^ and the Y-chromosome *attP* of y1 w*/Dp(2;Y)G, P{CaryP}attPY[30] to make DmY^L-tTA^. Transgenic female lethal strains were generated by microinjection and ΦC31 integration of pMM7-8-1 into the X-chromosome at *attP* sites y[1] w[*] P{y[+t7.7] = CaryIP}su(Hw)attP8 (BDSC #32233)[29] to create DmX^FL1-tTA^ or y[1]w[1118]pBac{y[+]-attP-9A}VK00006 (BDSC # 9726)[31] to create DmX^FL2-tTA^. Transgenic animals were isolated by crossing to FM6 balancers, then homozygosed by selecting non-balancer animals to generate a true breeding strain. DmX^FL12-tTA^ flies were created by isolating recombinant chromosome of DmX^FL1-tTA^ and DmX^FL2-tTA^ and screening for the presence of transgene in both locations of the X-chromosome.

### Fly viability assays

A desired number of male and virgin female flies were moved to new tubes containing media either in presence or absence of tetracycline and allowed to lay eggs for five days at 25°C and 12 h light protocol. After five days, adults were removed from the tubes and offspring were allowed to develop in the incubator. Adult flies were counted as they emerged from the pupae for a total of 15 days from the start of experiment.

### PCR Verification

Fly genomic DNA was isolated in a pool by grinding in 25 μl of "Squish Buffer" (10 mM Tris, 1 mM EDTA, 25 mM NaCl, 8 U/ml ProK (NEB P8107S)) per adult. ProK was heat inactivated at 98°C for 4 min. For transgene PCR screen, 1181 STSS males were pooled together as one sample and compared to 5 male and 5 female DMX^L-tTA^ flies in a separate pool of genomic DNA as positive control. The positive control samples were diluted in with STSS gDNA in the following ratios: 1:5, 1:25, 1:125, 1:625, and 1:3125. For each reaction, 1 μL of template gDNA was used in a 20 μL PCR reaction with primers that anneal within the transgene. The following primers were used for amplification of the transgene, fwd: 5’-GCCGCAGAATTCTCTCTATC-3’, rev: 5’-CTTAGCTTTCGCTTAGCGACG-3’; upd1, fwd: 5’-TGCAGGTGACCTGGGAATAG-3’, rev: 5’-GTGAGACCACTTGACCACAG-3’and kl-5, fwd: 5’-CGCGACGATAGACAGCGG-3’, rev: 5’-GAGAGCAATGCGCTCGTTGC-3’.

### Data analysis

All the experiments were performed with at least 2 and as high as 10 replicates. Raw offspring numbers from each experiment were converted into percent male/female and averaged across the replicates. Chi-squared test was performed to test difference between observed and expected sex ratio in different mating. Number of flies from each experiment across the different replicates was summed together then converted into percent male/female and used in the Chi-squared test. To test the effect of different parental male-female ratio on adult offspring numbers, One-way ANOVA was performed followed by Bonferroni’s post-hoc test. P-value <0.05 was considered significant. All the experimental data generated from the experiments are provided in S1 Data.

## Supporting information

S1 Fig An alternative approach of STSS. (**a**) Reproductive behaviour of X-linked Female Lethal construct used in female-lethal (FL-) STSS. (**b**) Mating scheme for producing non-transgenic males via FL-STSS. Combining non-transgenic females produced from the YL strain with adult male flies produced from the X^FL^ strain results in death of all offspring except for non-transgenic males. (**c**) Genetic design of FL construct. (**d**) Chromosomal location of FL constructs in two copy ‘FL12a-c’ flies. FL1 and FL2 have only one copy of the X-linked FL construct on their X-chromosome. (**e**) Proportion of male and female offspring generated from self-mating or outcrosses to wild-type (*w*^*1118*^) for DmX^FL1^, DmX^FL2^, and three independently generated DmX^FL12^ genotypes. Parental genotypes are indicated below the x-axis. Results are shown in the presence or absence of tetracycline. Numbers above bars indicate total number of progeny produced from at least three biological replicates. (**f**) Average number of adult males obtained from mating between different proportions of non-transgenic female flies obtained from DmYL^tTA^ in absence of tetracycline (‘XX*’) when combined with adult male DmX^FL12c^ flies in absence of tetracycline. Data represent mean numbers from 2 biological replicates with error bars showing standard deviation. Average numbers of females produced are indicated numerically above bars. Total numbers from all replicates are (from left to right): N = 460, N = 475, N = 692, N = 617. Numbers below x-axis indicate number of parental flies of each genotype. * indicates statistically significant difference from expected 50:50 male:female sex ratio (chi-squared test, p < .05). ** indicates a statistically significant difference between the +tet and -tet groups (chi-squared test, p<0.001).
(TIF)

S2 Fig Characterization of unexpected flies. Obtained female (♀*) from the final mating and male (♂*) from DmY^L-tTA^ mating in absence of tetracycline were assayed for the presence of X and Y-chromosome by amplifying an X-chromosome specific gene, upd1 and Y-chromosome specific gene, kl-5. L denotes 1 kb plus DNA ladder (Thermo Fisher Scientific, Waltham, MA).
(TIF)

S1 Data Source Data from the experiments. The Excel file (S1_Data.xlsx) contains results of each replicate mating experiment, including (columns: Replicate number, Tetracycline addition, Paternal parent genotype, Maternal parent genotype, number of male offspring surviving to adulthood, number of female offspring surviving to adulthood). Data are organized to correspond to each figure subpanel, as indicated by bold text in the file.
(XLSX)
