# Supplementary figures and images for: Genetic engineering of sex chromosomes for batch cultivation of non-transgenic, sex-sorted males
Source: PLoS Genet. 2020 Nov 2;16(11):e1009180. doi: 10.1371/journal.pgen.1009180 (PMC7660900; doi:10.1371/journal.pgen.1009180)

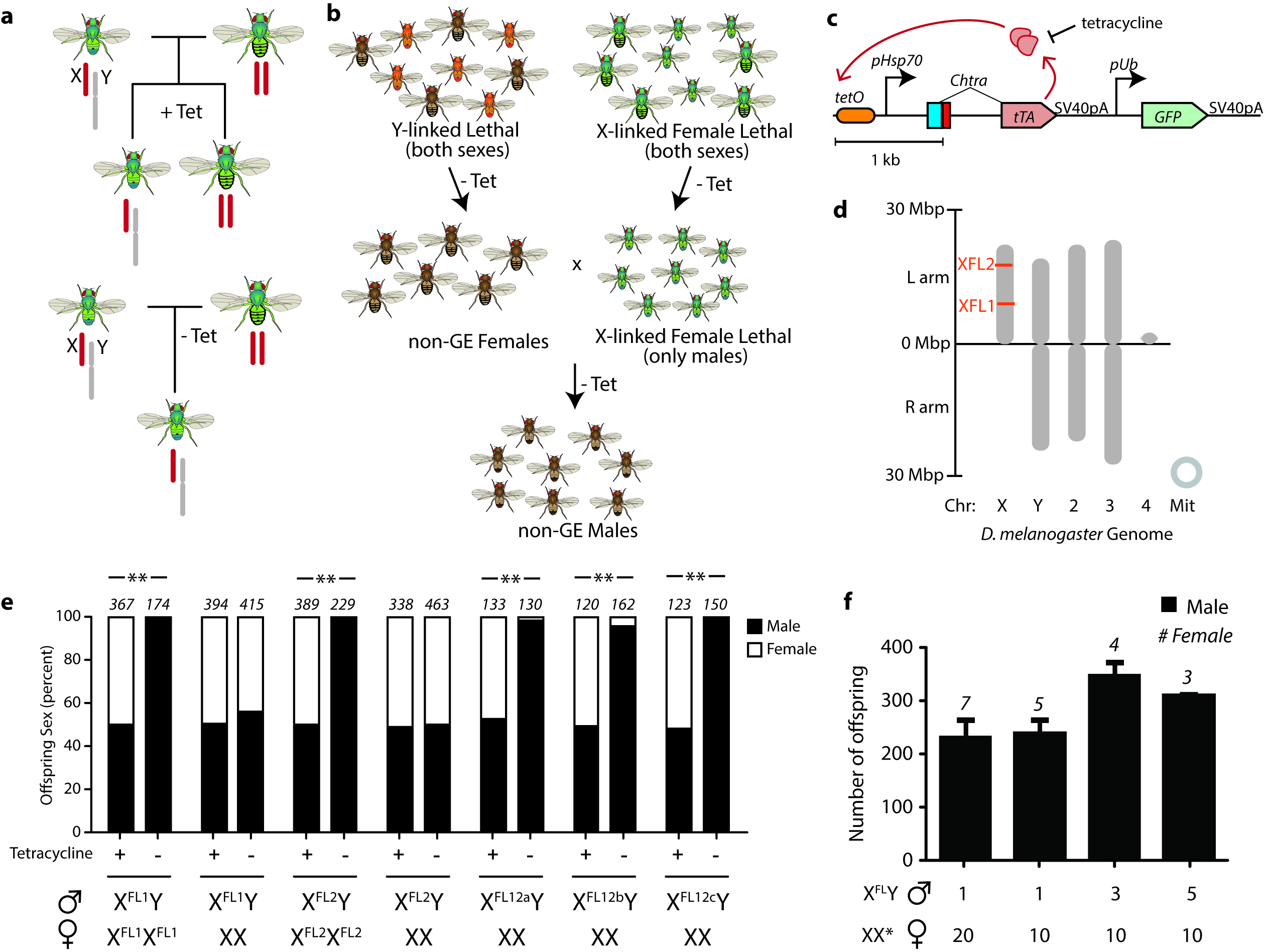

Supplement: S1 Fig — (a) Reproductive behaviour of X-linked Female Lethal construct used in female-lethal (FL-) STSS. (b) Mating scheme for producing non-transgenic males via FL-STSS. Combining non-transgenic females produced from the YL strain with adult male flies produced from the XFL strain results in death of all offspring except for non-transgenic males. (c) Genetic design of FL construct. (d) Chromosomal location of FL constructs in two copy ‘FL12a-c’ flies. FL1 and FL2 have only one copy of the X-linked FL construct on their X-chromosome. (e) Proportion of male and female offspring generated from self-mating or outcrosses to wild-type (w1118) for DmXFL1, DmXFL2, and three independently generated DmXFL12 genotypes. Parental genotypes are indicated below the x-axis. Results are shown in the presence or absence of tetracycline. Numbers above bars indicate total number of progeny produced from at least three biological replicates. (f) Average number of adult males obtained from mating between different proportions of non-transgenic female flies obtained from DmYLtTA in absence of tetracycline (‘XX*’) when combined with adult male DmXFL12c flies in absence of tetracycline. Data represent mean numbers from 2 biological replicates with error bars showing standard deviation. Average numbers of females produced are indicated numerically above bars. Total numbers from all replicates are (from left to right): N = 460, N = 475, N = 692, N = 617. Numbers below x-axis indicate number of parental flies of each genotype. * indicates statistically significant difference from expected 50:50 male:female sex ratio (chi-squared test, p < .05). ** indicates a statistically significant difference between the +tet and -tet groups (chi-squared test, p<0.001). (TIF) [file pgen.1009180.s001.tif]

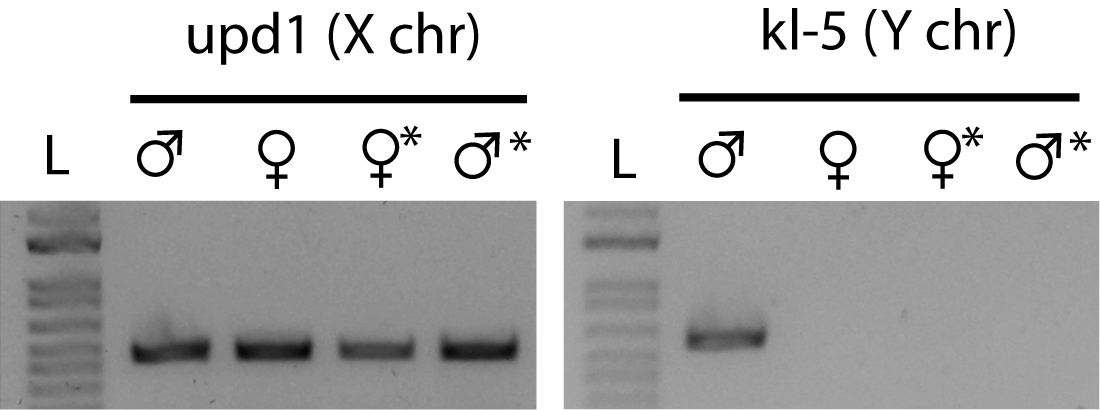

Supplement: S2 Fig — Obtained female (♀*) from the final mating and male (♂*) from DmYL-tTA mating in absence of tetracycline were assayed for the presence of X and Y-chromosome by amplifying an X-chromosome specific gene, upd1 and Y-chromosome specific gene, kl-5. L denotes 1 kb plus DNA ladder (Thermo Fisher Scientific, Waltham, MA). (TIF) [file pgen.1009180.s002.tif]
